# Insulin Receptor Substrate 2 Controls Insulin-Mediated Vasoreactivity and Perivascular Adipose Tissue Function in Muscle

**DOI:** 10.3389/fphys.2018.00245

**Published:** 2018-03-23

**Authors:** Alexander H. Turaihi, Wineke Bakker, Victor W. M. van Hinsbergh, Erik H. Serné, Yvo M. Smulders, Hans W. M. Niessen, Etto C. Eringa

**Affiliations:** ^1^Department of Physiology, Amsterdam Cardiovascular Sciences, VU University Medical Center, Amsterdam, Netherlands; ^2^Department of Internal Medicine, Amsterdam Cardiovascular Sciences, VU University Medical Center, Amsterdam, Netherlands; ^3^Department of Pathology and Cardiac Surgery, Amsterdam Cardiovascular Sciences, VU University Medical Center, Amsterdam, Netherlands

**Keywords:** insulin sensitivity, perivascular adipose tissue, insulin receptor substrate 2, microcirculation, endothelium

## Abstract

**Introduction:** Insulin signaling in adipose tissue has been shown to regulate insulin's effects in muscle. In muscle, perivascular adipose tissue (PVAT) and vascular insulin signaling regulate muscle perfusion. Insulin receptor substrate (IRS) 2 has been shown to control adipose tissue function and glucose metabolism, and here we tested the hypothesis that IRS2 mediates insulin's actions on the vessel wall as well as the vasoactive properties of PVAT.

**Methods:** We studied PVAT and muscle resistance arteries (RA) from littermate IRS2^+/+^ and IRS2^−/−^ mice and vasoreactivity by pressure myography, vascular insulin signaling, adipokine expression, and release and PVAT morphology. As insulin induced constriction of IRS2^+/+^ RA in our mouse model, we also exposed RA's of C57/Bl6 mice to PVAT from IRS2^+/+^ and IRS2^−/−^ littermates to evaluate vasodilator properties of PVAT.

**Results:** IRS2^−/−^ RA exhibited normal vasomotor function, yet a decreased maximal diameter compared to IRS2^+/+^ RA. IRS2^+/+^ vessels unexpectedly constricted endothelin-dependently in response to insulin, and this effect was absent in IRS2^−/−^ RA due to reduced ERK1/2activation. For evaluation of PVAT function, we also used C57/Bl6 vessels with a neutral basal effect of insulin. In these experiments insulin (10.0 nM) increased diameter in the presence of IRS2^+/+^ PVAT (17 ± 4.8, *p* = 0.014), yet induced a 10 ± 7.6% decrease in diameter in the presence of IRS2^−/−^ PVAT. Adipocytes in IRS2^−/−^ PVAT (1314 ± 161 μm^2^) were larger (*p* = 0.0013) than of IRS2^+/+^ PVAT (915 ± 63 μm^2^). Adiponectin, IL-6, PAI-1 secretion were similar between IRS2^+/+^ and IRS2^−/−^ PVAT, as were expression of pro-inflammatory genes (TNF-α, CCL2) and adipokines (adiponectin, leptin, endothelin-1). Insulin-induced AKT phosphorylation in RA was similar in the presence of IRS2^−/−^ and IRS2^+/+^ PVAT.

**Conclusion:** In muscle, IRS2 regulates both insulin's vasoconstrictor effects, mediating ERK1/2-ET-1 activation, and its vasodilator effects, by mediating the vasodilator effect of PVAT. The regulatory role of IRS2 in PVAT is independent from adiponectin secretion.

## Introduction

Insulin resistance, obesity and type 2 diabetes (DM2) are increasingly common risk factors for cardiovascular disease (Brownrigg et al., 2016). Resistance to insulin's vasodilator effects is characteristic of insulin resistant and type 2 diabetic subjects (Jiang et al., 1999; Okon et al., 2005), and has been shown to contribute to increased vascular resistance (Woerdeman et al., 2016), defects in organ perfusion and atherosclerosis (Rask-Madsen et al., 2010). As such, understanding and reversing defects in vascular insulin signaling contributes to prevention of cardiovascular complications of obesity and DM2.

After a meal, the physiological rise in plasma insulin levels induces pleiotropic effects on the muscle vasculature (Baron, 1993) to facilitate its access to myocytes. Insulin appearance in skeletal muscle interstitium is the rate limiting step for insulin's metabolic actions that promote glucose disposal (Yang et al., 1989), and therefore insulin access to the muscle interstitium contributes to whole-body insulin sensitivity (Kubota et al., 2011). In muscle microvessels, insulin can induce vasoconstriction through ERK1/2-dependent endothelin-1 (ET-1) production (Eringa et al., 2004) and vasodilatation through insulin receptor substrate1/2 (IRS1/2)- and Akt-dependent nitric oxide (NO) production (Montagnani et al., 2002; Meijer et al., 2015). While insulin's vasodilator actions predominate in normal conditions, insulin's vasoconstrictor effect is dominant in obesity and DM2, as a result of increased ET-1 production and decreased NO production (Lesniewski et al., 2008). The roles of IRS1 and−2 in insulin's vasoconstrictor actions have not been studied.

An important local regulator of insulin's vascular actions is perivascular adipose tissue (PVAT), which surrounds most vessels with an internal diameter >100 μm and consists of adipocytes, inflammatory cells and stem cells (Houben et al., 2012). Anatomical locations of PVAT include the aorta as well as the vascular networks of muscle and the heart (Mazurek et al., 2003; Verlohren et al., 2004; Meijer et al., 2015). PVAT serves as a source of adipokines which exert control over endothelial responses to insulin and other vasoactive stimuli (Greenstein et al., 2009). Understanding the signaling pathway between PVAT and the vasculature potentially uncovers new therapeutic targets to treat disorders such as hypertension and DM2. Resistance to insulin-induced vasodilatation is better understood when taking the continuous interplay between PVAT and the vasculature into consideration. PVAT secretes adiponectin which signals through AMP-activated protein kinase (AMPK) and Akt to stimulate NO production, uncovering insulin-mediated vasodilation (Meijer et al., 2015; de Boer et al., 2016). We have previously shown that PVAT from db/db mice secretes less adiponectin and fails to induce insulin-mediated vasodilation when compared to wild-type PVAT (Meijer et al., 2013). Thus, qualities inherent to PVAT are important in endothelial reactivity to insulin.

Genetic mutations in IRS1 and IRS2 have been associated with DM2 and impaired vascular function (Jiang et al., 1999; Esposito et al., 2003; Bodhini et al., 2007). Aside from its role in insulin signal transduction (Sun et al., 1995), IRS2 functions independently in insulin growth factor-1 (IGF-1) and anti-inflammatory cytokine signaling (O'Connor et al., 2007). Mice lacking IRS2 show insulin resistance and beta-cell failure, resulting in peripheral insulin resistance and DM2 after 8–10 weeks of age (Kubota et al., 2000; Withers et al., 2014). Importantly, IRS2 also regulates endocrine functions of adipose tissue, inhibiting fatty acid synthesis (Previs et al., 2000). Moreover, insulin's effects on adipose tissue have been shown to control glucose uptake in muscle (Abel et al., 2001). Despite the recognition that IRS2 plays an important role in glucose homeostasis and adipose tissue function, the role of IRS2 in PVAT function is unknown.

The aim of this study was to elucidate the role of IRS2 in control of muscle perfusion by insulin and as well as the mechanisms involved. To this end, we used the *ex vivo* pressure myograph to investigate effects of insulin on muscle resistance arteries (RA) in the absence and presence of PVAT.

## Materials and methods

### Animals

Animal experiments were performed in accordance with the European Community Council Directive 2010/63/EU for laboratory animal care and the Dutch Law on animal experimentation. The experimental protocol was validated and approved by the local committee on animal experimentation of the VU University Medical Center. Male C57Bl/6NCrl mice (further indicated as Bl6) were bred in-house (obtained from Harlan, Horst, the Netherlands). Male IRS2^+/+^ and IRS2^−/−^ mice, on a hybrid background of the Bl6 and SV129 mice strains (Jackson Laboratories, Maine, USA), were obtained by heterozygous breeding. PCR was used to confirm the genotype of the mice as described (Withers et al., 1999) with primers: *5*′-*GTCATCAGGACATAGCGTTGG-3*′*,5*′-*CTTGGCTACCATGTTGTTATTGTC-3*′*, 5*′-*AGTTCTGGAGGTTTACTTTCCTAG-3*′. Sv129 mice (Jackson laboratories, Maine, USA) were used to check for differences in genetic background in insulin responses. Mice were housed in standard cages and were fed chow diet and water *ad libitum*. Mice were sacrificed by isoflurane overdose after overnight fasting at 8 weeks age.

### Vasoreactivity experiments

First-order RA from the gracilis muscle were isolated from lean Bl6, IRS2^+/+^ and IRS2^−/−^ mice after an overnight fast. PVAT surrounding the RA of Bl6, IRS2^+/+^ and IRS2^−/−^ mice was isolated from the section of the RA between its origin at the femoral artery and its first major side branch within the gracilis muscle as described (Meijer et al., 2013). RA's were cannulated in a pressure myograph and studied at a pressure of 80 mmHg and a temperature of 37°C in K-MOPS buffer with a KCl concentration of 25 mM, as described previously (Meijer et al., 2013).

RA's were randomly assigned to incubation either without PVAT (*n* = 9) or with IRS2^+/+^ (*n* = 9) and IRS2^−/−^ PVAT (*n* = 10) with approximately equal amounts PVAT used in each condition. Preconstriction of 40% was achieved with KCl, the inner diameter of RA's was recorded to determine baseline diameter and diameter changes induced by four concentrations of insulin (0.01, 0.1, 1, and 10 nM) (Novorapid; Novo Nordisk, Bagsværd, Denmark), each exposure being for 30 min. The three lowest insulin concentrations of insulin are within the physiological range, with the third concentration (1 nM) corresponding to postprandial levels, whereas the fourth concentration is pharmacological. Smooth muscle function was tested as KCl-induced vasoconstriction and only vessels which showed a constriction of >40% of their maximal diameters were used for experiments. Endothelial integrity was determined by measuring responses to the endothelium-dependent vasodilator acetylcholine 1^*^10^−7^ M (ACh) and the end of each experiment, an RA failing to achieve at least 10% vasodilation to ACh were excluded from all analyses. The role of ET-1 was assessed by pre-treatment for 30 min with the non-selective ET-1 receptor antagonist (PD142893: 10 μM, Kordia, Leiden, the Netherlands) before the addition of insulin. The vasomotor response to insulin was expressed as a percentage of the baseline diameter, i.e., the vessel diameter immediately before addition of the first concentration of insulin.

### Western blot

Protein analyses were performed by Western blotting, as described (Meijer et al., 2013). Segment of RA from IRS2^+/+^ and IRS2^−/−^ mice were exposed to solvent or to insulin for 15 min at 37°C. In order to study the effects of PVAT on the RA, IRS2^+/+^ and IRS2^−/−^ PVAT were isolated from overnight fasted mice and then incubated in 100 μl MOPS buffer with 1% of *bovine serum albumin (*BSA). PVAT samples were stimulated with either solvent or 10 nM insulin for 30 min in a 96 well-plate. Thereafter, freshly isolated femoral artery segments from fasted 8 week old Bl6 mice were added to the PVAT wells and incubated at 37°C for 15 min. Femoral artery segments were used in order to obtain appropriate amounts of protein for Western blotting. The artery segments were snap frozen in liquid nitrogen and savedat −80°C till further analysis. The protein lysates were stained with a specific primary antibody against Ser 473 phosphorylated Akt (antibodies obtained from Cell Signaling Technology, Boston, MA, USA) and were visualized with a chemiluminescence kit (GE Healthcare, Diegem, Belgium). A specific primary antibody against ERK1/2 (1:1,000; New England Biolabs, Ipswich, USA) was used to examine ERK1/2 activation. Differences in phosphorylated protein of Akt at ser 473 were adjusted for differences in the total Akt protein staining.

### Adipokine secretion

PVAT-conditioned media were prepared and the amount of secreted adipokines were quantified using the mouse magnetic-bead adipokine milliplex multianalyte ELISA kit (Millipore, Amsterdam, the Netherlands) and detected using the Luminex system. Freshly isolated PVAT from fasted IRS2^+/+^ (*n* = 7) and IRS2^−/−^ (*n* = 5) mice in amounts of comparable size were incubated in 100 μl MOPS buffer with 1% of BSA and stimulated with either solvent or 10 nM insulin at 37°C for 45 min in a 96 well-plate. The conditioned media were snap frozen in liquid nitrogen and saved at −80°C till further analysis. Adiponectin, IL-6, Leptin, MCP-1, PAI-1 (Total), Resistin, and TNF-α concentrations in the conditioned media were measured in duplicate and averaged. Data are corrected for the PVAT weight that was used in the incubation.

### Real time quantitative polymerase chain reaction (qRT-PCR)

Total RNA was extracted from PVAT using a miRCURY RNA isolation kit (Exiqon). PVAT RNA was reversely transcribed and amplified using Ovation PicoSL WTA System V2 (Nugen). Quantitative PCR was performed using a commercial SYBR green mastermix (Biorad) and specific primers (available upon request) for pro-inflammatory genes (TNF-α and CCL2) and adipokines (adiponectin, leptin, and endothelin-1). IRS1 expression was evaluated to check for compensation to IRS2 knockout. Data were corrected for the geometric mean of ribosomal protein S15 (*Rps15*) and expressed relative to the wild type PVAT.

### Histology

PVAT surrounding the RA was excised together with part of the underlying muscle and stored overnight in buffered formaldehyde (4%) and embedded in paraffin. For histochemical analysis, slices with a thickness of 5 μm were dewaxed, rehydrated and stained with hematoxylin and eosin (H&E). The cross-sectional area of adipocytes was analyzed in blinded fashion using Image J software.

### Blood pressure and heart rate measurements

Blood pressure and heart rate of IRS2^+/+^ and IRS2^−/−^ were determined under stress-free conditions using radio telemetry as described (Aman et al., 2012).

### Statistical analysis

Values are expressed as mean ± SEM. Steady-state responses are reported as mean change from baseline (percentages) ± SEM. Differences in insulin-induced vasoreactivity were performed using two-way ANOVA. Tukey *post-hoc* test was where appropriate. Differences in IHC analyses and in protein phosphorylation as found by Western blot were determined using unpaired student *t*-test. Phosphorylation was expressed as the fold increase over the unstimulated controls, assigning a value of 1 to the control. qRT-PCR data was log2 transformed before statistical analysis. Differences with *p* < 0.05 were considered statistically significant. Analyses were performed with GraphPad Prism 6.0 (GraphPad software, San Diego, CA, USA).

## Results

### General characteristics of IRS2^+/+^ and IRS2^−/−^mice

IRS2^+/+^ and IRS2^−/−^littermates had similar body weights (23 ± 1 and 24 ± 1 g, respectively, Table 1). IRS2^−/−^ mice had higher non-fasting levels of plasma insulin (9 ± 1 vs. 20 ± 2 μU/ml, *p* < 0.01) and exhibited fasting hyperglycemia (5.3 ± 0.57 vs. 12.6 ± 2.7 mMol/l, *p* < 0.05) than IRS2^+/+^. IRS2^−/−^ mice showed a small decrease in blood pressure compare to their IRS2^+/+^ littermates (Table 1).

**Table 1:** General characteristics of IRS2-deficient and wild-type littermates at 8 weeks of age.

**General characteristics**	**IRS2^+/+^**	**IRS2^−/−^**	***P***
Body weight (g)	23 ± 1	24 ± 1	0.84
Fasting blood glucose (mMol/l)	5.3 ± 0.5	12.6 ± 2.7	<0.05
Non-fasting blood glucose (mMol/l)	8.7 ± 0.8	12.2 ± 0.9	<0.01
Non-fasting blood insulin (μ*U*/*ml*)	9 ± 1	20 ± 2	<0.01
MAP (mm Hg)	115 ± 3	101 ± 5	<0.05
SBP (mm Hg)	127 ± 3	110 ± 6	<0.05
Heart rate (beats per minute)	589 ± 11	553 ± 19	0.08

*IRS2^−/−^ had similar body weights and higher levels of insulin and glucose than the IRS2^+/+^ mice. MAP, mean arterial pressure, SBP, systolic blood pressure. Data represent mean ± SEM, IRS2^+/+^N = 10, IRS2^−/−^N = 7*.

### Insulin-mediated vasoreactivity in IRS2^+/+^ and IRS2^−/−^ muscle resistance arteries

**Figure 1:**
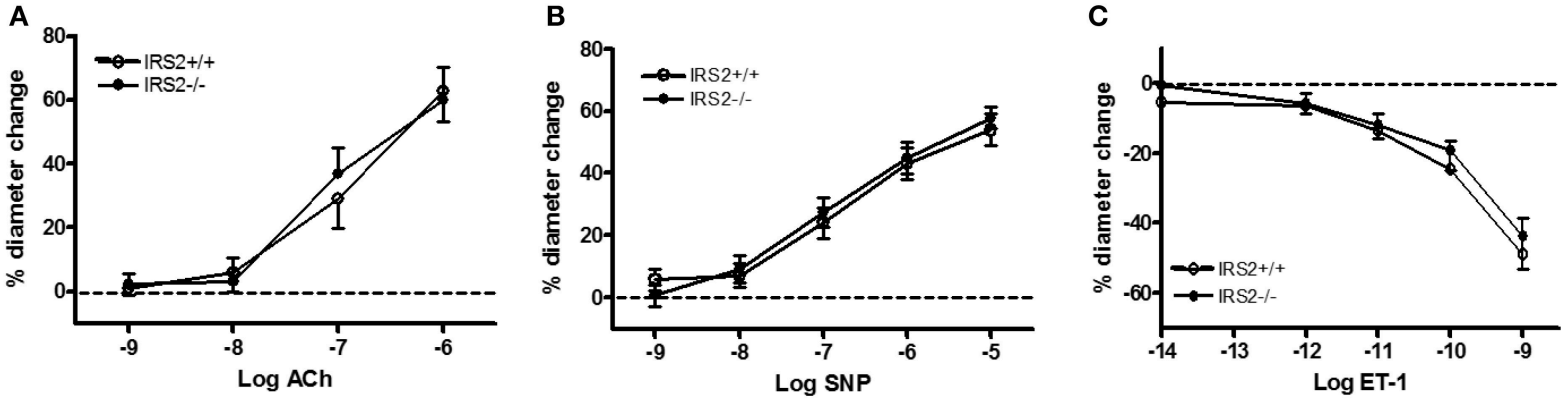
Vasoregulatory responses in resistance arteries of IRS2^−/−^ mice without PVAT. In all experiments on tissues of IRS2^−/−^ mice, their IRS2^+/+^ littermates were the control strain. Acetylcholine, ACh **(A)**; sodium nitroprusside, SNP **(B)** and ET-1 **(C)** were similar in IRS2^+/+^ (°, *n* = 5) and IRS2^−/−^ resistance arteries (•, *n* = 6).

**Figure 2:**
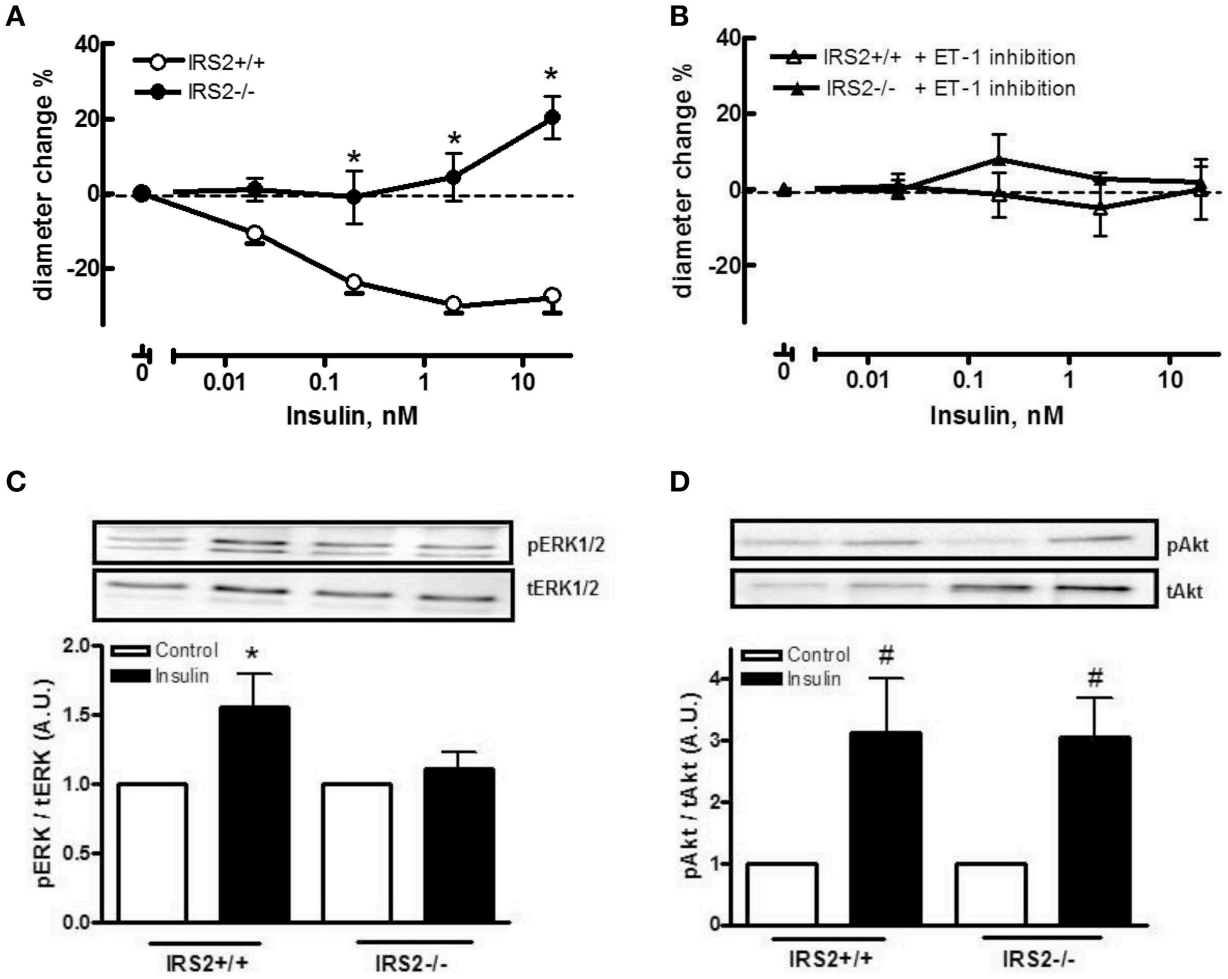
IRS2 mediates insulin-stimulated ERK1/2-ET-1 activity in muscle resistance arteries without PVAT. Responses of IRS2^+/+^ and IRS2^−/−^ RA to insulin **(A)** and during endothelin receptor blockade using PD142893 (10ulMol/l) **(B)**, *n* = 5, ^*^p < 0.01 vs. IRS2^+/+^. **(C,D)**: IRS2 deletion decreases insulin-stimulated phosphorylation of ERK1/2, but not of Akt in muscle RA. ERK1/2 and Akt phosphorylation were compared between insulin stimulation (2 nM) (black bars) and the control situation (white bars) in segments of the gracilis RA from IRS2^+/+^ (*n* = 8) and IRS2^−/−^ (*n* = 9) mice, ^*^p < 0.05; ^#^*p* < 0.01 vs. control.

To gain insight into the role of IRS2 in insulin-dependent vasoreactivity in muscle, we first examined the reactivity of IRS2^+/+^ and IRS2^−/−^ gracilis muscle RA obtained from IRS2^+/+^ and IRS2^−/−^ mice in response to insulin and other vasoactive molecules.IRS2^+/+^ and IRS2^−/−^RA showed normal endothelial and smooth muscle function, as reflected by responses to acetylcholine (Figure 1A), sodium nitroprusside (Figure 1B), and exogenous ET-1 (Figure 1C). The maximal diameter of IRS2^−/−^ RA was reduced compared to IRS2^+/+^ RA (111 ± 6 vs. 127 ± 5 micron, *P* < 0.05), and basal vascular tone in the presence of 25 mM potassium was 34 ± 2% in IRS2^+/+^ and 27 ± 4% in IRS2^−/−^ RA (*P* = 0.11). Unexpectedly, insulin induced vasoconstriction in isolated IRS2^+/+^RA, which was inhibited by the non-selective ET-1 receptor antagonist PD142893 (Figures 2A,B). This confirms the critical role of endothelin-1 ininsulin's vasoconstrictor effects, and shows a decreased contribution of NO to insulin-mediated vasoreactivity in IRS2^+/+^ RA. In IRS2^−/−^ mice, no vasoconstrictor response was observed during exposure of the isolated arteries to insulin, nor in the presence of the ET-1 receptor antagonist (Figure 2B). Mechanistically, the insulin-induced phosphorylation of ERK1/2 was impaired in IRS2^−/−^ RA compared to IRS2^+/+^ RA (Figure 2C), whereas insulin-stimulated activation of Akt was similar (Figure 2D). This indicates that the stimulation of ERK1/2-ET-1 activity by insulin requires IRS2.

The observed insulin-induced vasoconstriction of isolated IRS2^+/+^ RA contrasts with the response of C57Bl/6 mice (Meijer et al., 2013), which vasodilate in response to insulin during ET-1 receptor inhibition and during incubation with PVAT of lean Bl6 mice (data not shown). As the IRS2 mice used in this study are bred on a mixed background of the mouse strains C57Bl/6 and SV129, we tested whether the Sv129 background caused the vasoconstrictor response by studying insulin responses of isolated RA from SV129 mice. Insulin indeed induced vasoconstriction in arteries from SV129 mice, while it had no net effect on the diameter of C57Bl/6 RA (Figure S1A). Endothelial function (response to acetylcholine-mediated vasodilation) was not different between the two strains (Figure S1B). Collectively, arteries from different mouse strains can display different responses to insulin. For this reason and to facilitate comparability with other experiments, we proceeded by studying the effect of PVAT obtained from IRS2^+/+^ and IRS2^−/−^ mice on RA's obtained from C57Bl/6 mice. RA's from C57/Bl6 mice had a maximal diameter of 137 ± 5 microns.

### PVAT from IRS2^+/+^ but not IRS2^−/−^mice uncovers insulin-induced vasodilation in resistance arteries

**Figure 3:**
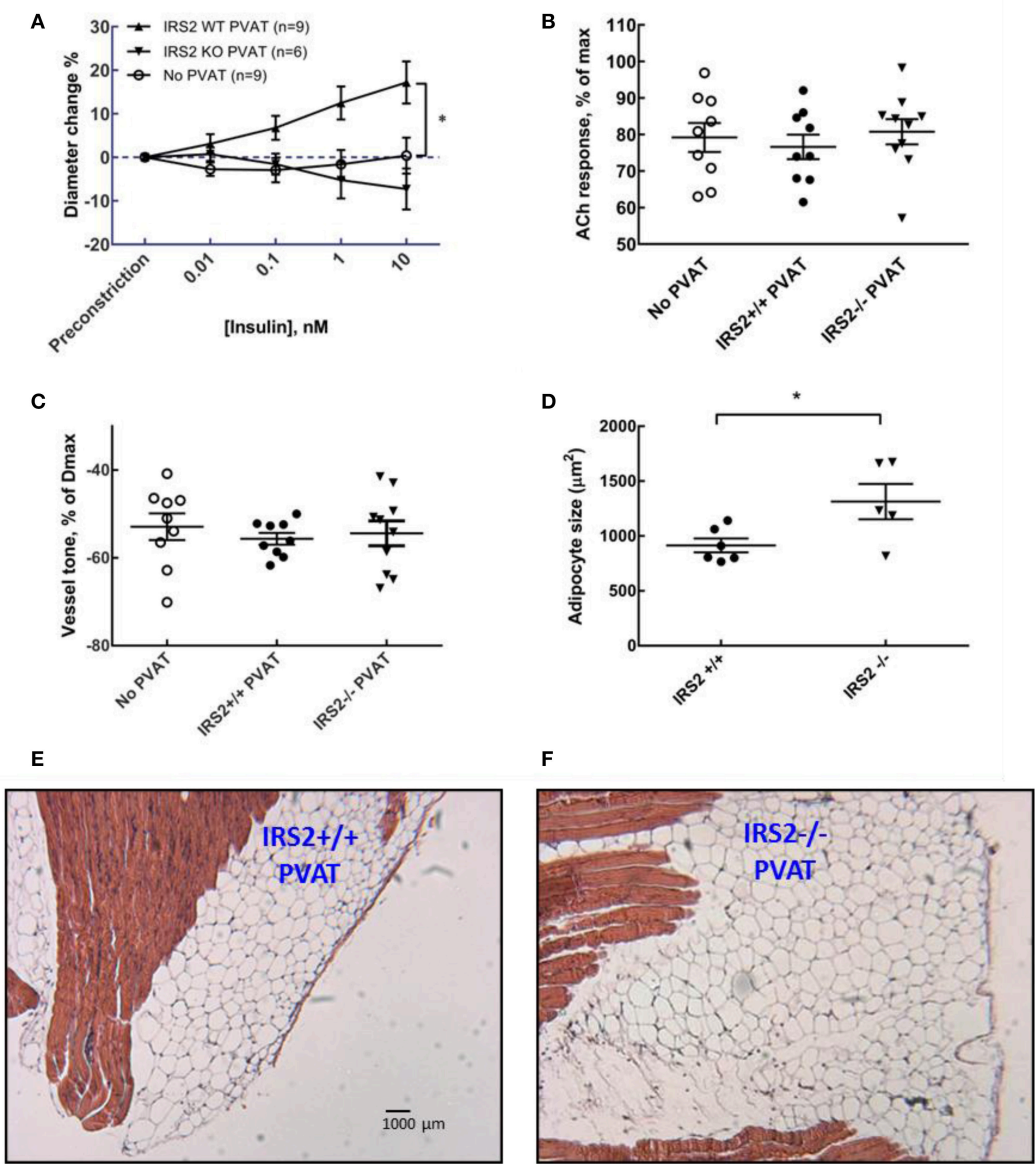
IRS2^−/−^ PVAT does not uncover insulin-mediated vasodilation. **(A)** Arteries obtained from C57/Bl6 mice were incubated with PVAT from IRS2^+/+^ and IRS2^−/−^ littermates and stimulated with increasing doses of insulin. Incubation with IRS2^+/+^ PVAT, allowed the artery to dilate in reaction insulin in dose-dependent manner (17.2 ± 4.9% dilation in response to highest (insulin), *p* < 0.0001, *N* = 9,) while arteries that were incubated with IRS2^−/−^ PVAT or were deprived of PVAT did not react to insulin (−7.3 ± 4.7%, *N* = 10 and 0.4 ± 4.1%, *N* = 9, respectively; *p* = 0.17). Data are presented as mean ± SEM, tested with two-way ANOVA with Tukey *post-hoc* correction. **(B)** At the end of the experiment, endothelial integrity was examined by stimulating the arteries with acetylcholine. All the arteries that were included in the analyses in **(A)** had at least 60% dilation in response to acetylcholine—cutoff point to rule out endothelialdamage. **(C)** PVAT did not affect the percentage of arteriolar preconstriction in the organ bath. **(D)** Adipocyte size in IRS2^+/+^ PVAT (412.1 ± 58.7 μm) are smaller (*p* = 0.03) than adipocytes in IRS2^−/−^ PVAT (914.7 ± 63. 2 μm). Data are presented as mean ± SEM, tested with unpaired *t*-test. **(D,E)**: Morphology of IRS2+/+ and IRS2^−/−^ PVAT at the site of the gracilis resistance artery. **(E,F)** are at the same magnification. M: muscle.

To study the interaction between PVAT and insulin-induced vasoreactivity of isolated muscle RA's, PVAT of IRS2^+/+^ and IRS2^−/−^ littermates was co-incubated with RA's in the pressure myograph for 45 min. Insulin (10 nM) induced vasodilation in the presence of IRS2^+/+^ PVAT (17.2 ± 4.9%, *N* = 9; Figure 3A). On the other hand, insulin failed to induce diameter change in arteries incubated with IRS2^−/−^ PVAT (−7.3 ± 4.7%, *N* = 10) or without the presence of PVAT (0.4 ± 4.1%, *N* = 9). Baseline vessel tone was not different in RA's (*n* = 28) in the presence of either IRS2^+/+^ PVAT orIRS2^−/−^ PVAT nor was acetylcholine-induced vasodilation (Figures 3B,**C**).

### Adipocyte size is increased in PVAT of IRS2^−/−^ mice

**Figure 4:**
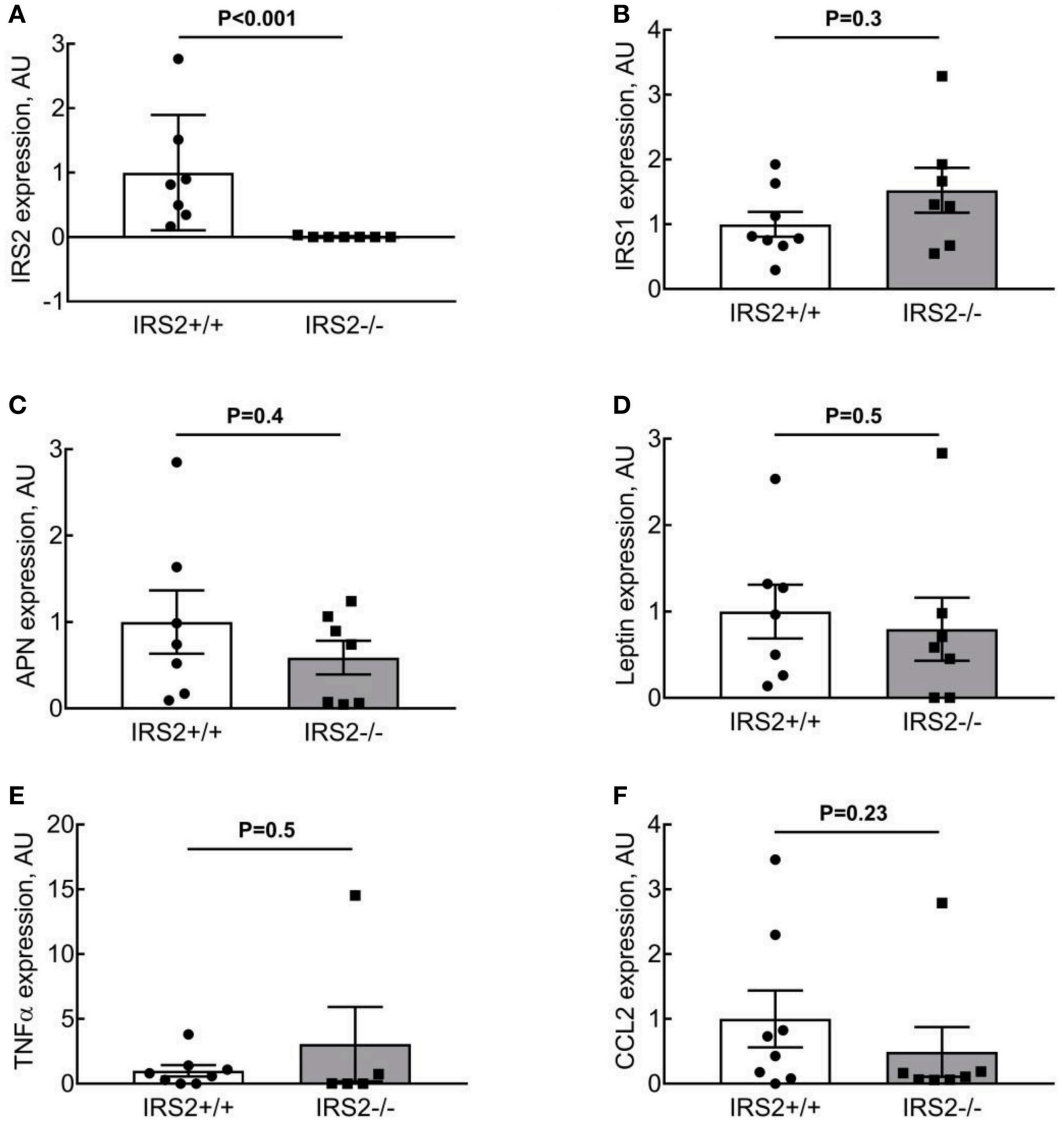
IRS2^−/−^PVAT does not show increased inflammatory gene expression. **(A,B)** IRS expression in IRS2^−/−^ mice. **(C,D)** IRS2 deficiency does not alter adiponectin and leptin expression in PVAT. **(E,F)** IRS2 deficiency does not enhance inflammatory gene expression in PVAT. Data were log-transformed prior to statistical analysis as described in section Methods. APN, Adiponectin.

To quantify the effects of IRS2 deletion on the morphology and inflammatory status of PVAT, adipocyte area and macrophage infiltration were investigated. Adipocyte area was significantly (*p* = 0.0013) larger in IRS2^−/−^ PVAT (1,314 ± 161 μm^2^) compared to IRS2^+/+^ (915 ± 63 μm^2^) (Figure 3D). Leukocyte infiltration assessed in tissue slices was not quantifiable in PVAT of both genotypes. IRS2 mRNA levels in PVAT were confirmed to be absent in IRS2^−/−^ PVAT (Figure 4A). To check for compensation for IRS2 knockout, we measured IRS1 mRNA expression and we found no differences between IRS^+/+^ and IRS^−/−^ PVAT (Figure 4B).

### Adiponectin expression and secretion from IRS2^−/−^ PVAT are similar to IRS2^+/+^ PVAT

**Figure 5:**
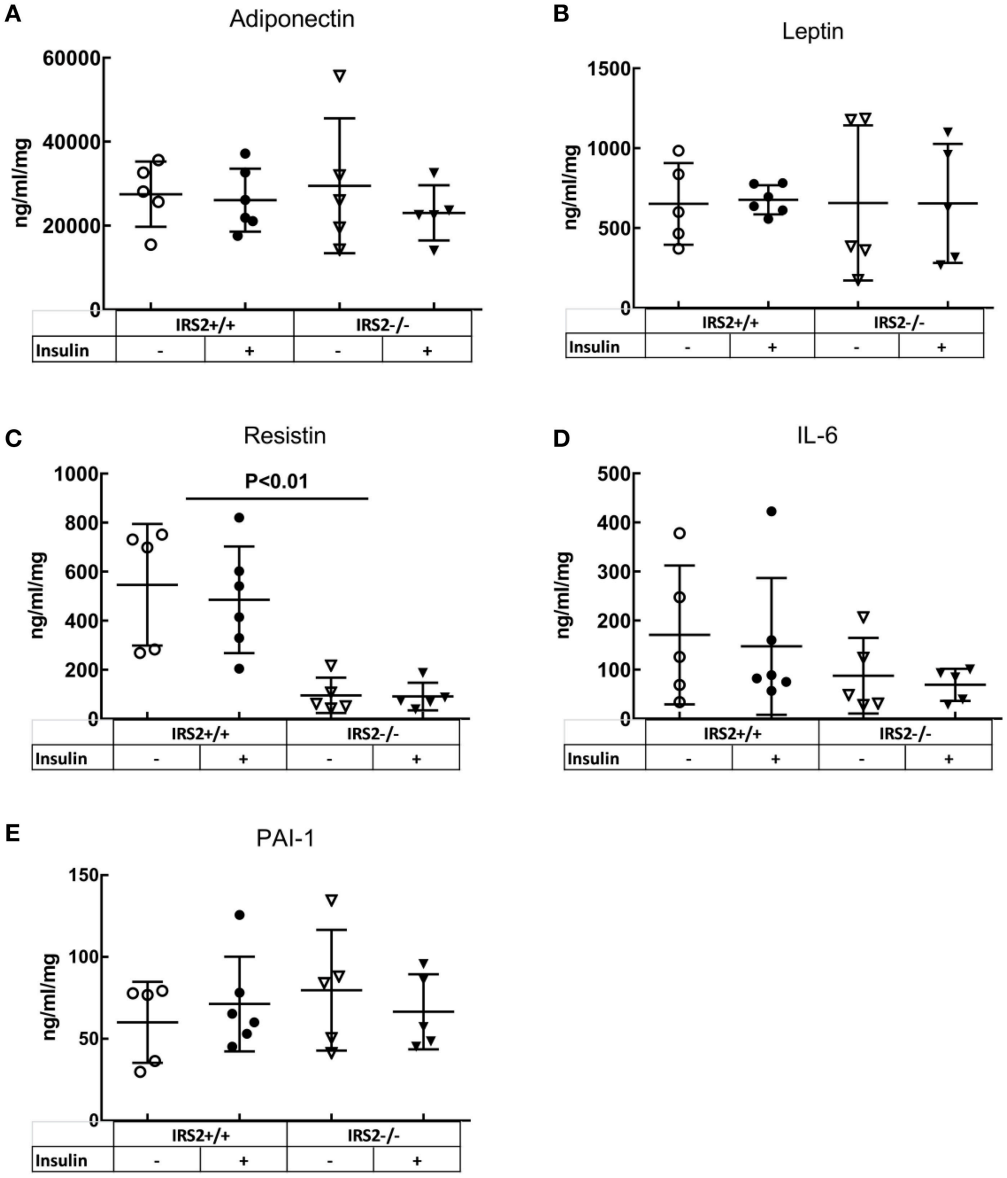
IRS2^−/−^ PVAT secretes equal amounts of Adiponectin compared to wild-type PVAT. There are no significant differences between IRS2^+/+^ and IRS2^−/−^ PVAT in **(A)** adiponectin, **(B)** IL6, **(D)** Leptin, **(E)** PAI-1. **(C)** Resistin secretion is decreasedin IRS2^−/−^ PVAT. Insulin stimulation did not affect the secretion of the measured adipokines. The amounts of MCP-1 and TNF-α were below the detection limitof the assay.

To elucidate the mechanisms involved in the interaction of intramuscular PVAT with insulin-induced vasoreactivity, we examined the role of several secreted adipokines in this interaction. Adiponectin secretion was similar between IRS2^+/+^ and IRS2^−/−^ PVAT conditioned media (Figure 5A), as was Adiponectin mRNA in PVAT between IRS2^+/+^ and IRS2^−/−^ mice (Figure 4C). Despite the increase in adipocyte size, leptin expression was similar between IRS2^+/+^ and IRS2^−/−^ PVAT (Figure 4D). Other adipokines and inflammatory markers were also studied. Levels of TNF-α and MCP-1 were undetectable in PVAT-conditioned medium (data not shown). Moreover, TNF-α mRNA levels were low in IRS2^+/+^ and IRS2^−/−^ PVAT (Figure 4E) while mRNA levels of CCL2 were similar between IRS2^+/+^ and IRS2^−/−^ PVAT (Figure 4F). There were no differences between IRS2^+/+^ and IRS2^−/−^ in the amounts of secreted PAI-1, IL-6, Leptin (Figures 5B,D,E). Unexpectedly, we found that IRS2 deficiency decreases resistin secretion by PVAT (Figure 5C), demonstrating that IRS2 deficiency does alter the secretory function of PVAT.

### Normal phosphorylation of Akt in response to insulin in arteries incubated with IRS2^−/−^ PVAT

**Figure 6:**
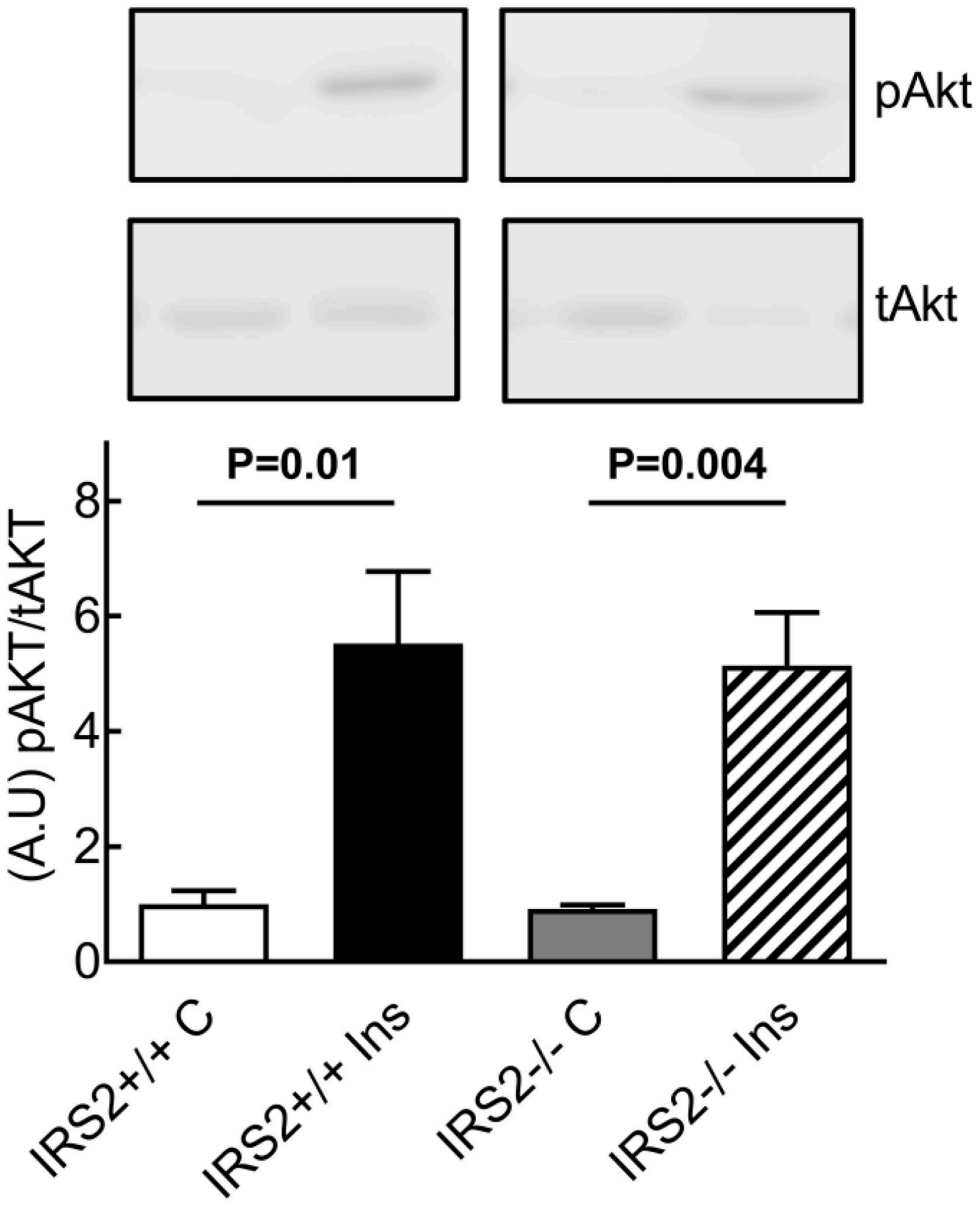
Normal insulin-stimulated phosphorylation of Akt in arteries incubated with IRS2^−/−^ PVAT. Insulin stimulation for 15 min resulted in strong Akt phosphorylation with no differences between incubation with IRS2^−/−^or wildtype PVAT.

To further study the insulin-induced vasodilator pathway, which is mediated through Akt and eNOS phosphorylation, phosphorylation of Akt was studied in the presence of IRS2^+/+^ and IRS2^−/−^ PVAT. The phosphorylation of Akt in femoral artery segments of C57Bl/6 mice was significantly increased after insulin stimulation in the presence of IRS2^+/+^ as well as IRS2^−/−^ PVAT (Figure 6).

## Discussion

**Figure 7:**
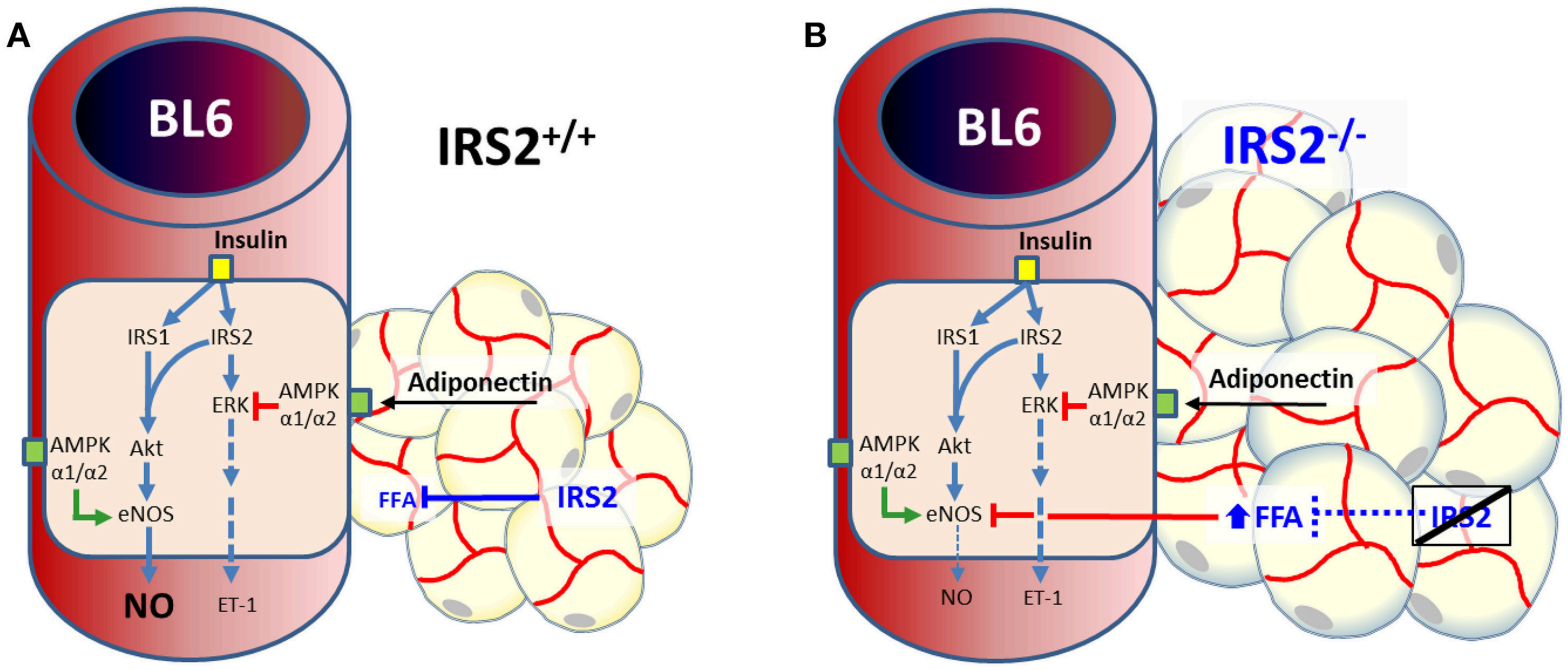
Dual role of IRS2 in regulation of insulin-induced vasoreactivity in muscle. **(A)** In health, insulin induces vasodilation by activating its receptor, resulting in phosphorylation and activation of insulin receptor substrates (IRSs) 1 and 2.both IRS isoforms are involved inAkt and endothelial Nitric Oxide Synthase (eNOS) activation, while IRS2 mediates insulin-induced activation of ERK1/2. Perivascular adipose tissue (PVAT) secretes vasodilator adipokines such adiponectin that reduce endothelin (ET-1) secretion (de Boer et al., 2016) and enhance NO production in endothelial cells through AMPK (Eringa et al., 2010). **(B)** IRS2-deficient PVAT fails to uncover insulin-mediated vasodilation in muscle arteries. Probable mechanisms aredirect mediators that are released from PVAT that interact with eNOS to reduce NO production, such as free fatty acids (FFA) (Previs et al., 2000).

The main findings of this study are fourfold: (1) IRS2 in the vessel wall of muscle RA is more involved in the ERK1/2-Endothelin-1 pathway than in the Akt-NO pathway of insulin signaling, (2) IRS2^−/−^ PVAT loses its ability to induce insulin-mediated vasodilation in skeletal muscle RA, (3) this is accompanied by increased adipocyte area in IRS2^−/−^ PVAT with no signs of increased inflammation, (4) adiponectin secretion is similar between IRS2^−/−^ PVAT and wildtype PVAT. A graphical summary of our findings is depicted in **Figure 7**.

It has been shown that IRS2 is a major IRS isoform expressed in endothelial cells (Kubota et al., 2003). In their important work, Kubota et al. (2003) showed that mice lacking IRS2 in endothelial cells had impaired insulin-induced eNOS phosphorylation. This resulted in a reduced insulin-mediated microvascular recruitment and a decrease in muscle glucose uptake. Insulin induces a strong vasoconstriction in the IRS2^+/+^ arteries which is completely dependent on ET-1 (Figure 2). We found out that this is due the difference in mouse strains as the Sv129-strain is less sensitive to insulin-mediated NO production in the vascular wall (Supplemental Figure S1). Importantly, the arteries that displayed vasoconstriction were in an experimental setup that is deprived of IRS2^+/+^ PVAT. Nevertheless, this insulin-induced vasoconstriction observed in IRS2^+/+^ RA is different than our earlier observations in human and Bl6 arteries that did not show insulin-induced vasoconstriction. Moreover, these arteries showed insulin-mediated vasodilation after preincubation with PVAT from lean mice. This is why we decided to proceed with our experimental set-up using RA obtained from Bl6 mice and constantly comparing the IRS2^−/−^ PVAT with PVAT obtained from their wildtype littermates. We show in our study that the absence of IRS2 in PVAT is sufficient to abrogate the PVAT-assisted insulin-mediated vasodilation of skeletal muscle RA despite the fact that these arteries were functionally normal (Figure 3A). In their seminal work, Abel et al. showed that alterations in adipocyte inherent characteristics resulted in a decreased muscle glucose uptake (Abel et al., 2001). This means that adipose tissue is capable of (in)directly altering the capacity of the body to regulate glucose homeostasis. In their study, however, the authors did not specifically examine the function of PVAT in these mice.

Kubota et al. have reported that the expression of IRS2 in endothelial cells is reduced by high fat diet in mice (Kubota et al., 2003). Conditions of calorie excess lead to a low-grade inflammation and production of pro-inflammatory cytokines (TNF-α and IL-6) from adipose tissue (Yudkin et al., 1999, 2005) which inhibits adiponectin secretion (Tilg and Moschen, 2006). Indeed, PVAT anticontractile properties are lost due to inflammation (Greenstein et al., 2009; Withers et al., 2011). After we observed that IRS2^−/−^ PVAT has lost its vasodilatory capacity, we looked at inflammatory signs in this tissue. Literature has shown that, as fat mass increases in obesity, the size of adipocytes increases. In our study, adipocytes in IRS2^−/−^ PVAT were larger than IRS2^+/+^ PVAT (Figure 3D). PVAT hypertrophy in IRS2^−/−^ mice may be caused by decreased resistin expression, as resistin stimulates lipolysis (Ort et al., 2005). (Mita et al., 2011) showed that IRS2-deficient macrophages accumulate in the vascular wall; eventual accumulation in PVAT is expected since PVAT is vascularized. In our study, however, we failed to show signs of inflammation in IRS2^−/−^ PVAT. First, macrophage staining in PVAT samples was not quantifiable because of the small size of PVAT samples and low prevalence of leukocytes (data not shown). Second, there were no differences in IL-6 secretion from IRS2^−/−^ and wildtype PVAT and the concentration of TNF-α was low (Figure 5). Third, we did not find differences in CCL2 and TNF-α mRNA expression levels between IRS2^−/−^ and wild-type PVAT. It would be interesting to quantify markers of inflammation in IRS2^−/−^ PVAT obtained from older mice to study age related changes in PVAT in conditions of IRS2 deficiency. However the functional defects of IRS2-deficient PVAT are unlikely to be caused by exaggerated inflammation.

Adiponectin is an important modulator of many metabolic processes and has been shown to increase insulin sensitivity and improve vascular function (Funahashi et al., 1999; Yokota et al., 2000; Laakso, 2001). The concentration of adiponectin is inversely associated with DM2 (Duncan et al., 2004; Luo et al., 2010). Adiponectin binds its receptor and activates AMPKα1α2 which eventually stimulates Akt phosphorylation and NO production (Eringa et al., 2006; de Boer et al., 2016). In the current study, however, we did not see differences in the secretion of adiponectin (Figure 5A) or expression levels thereof in IRS2^−/−^ and IRS2^+/+^ PVAT (Figure 4C). To confirm this, Akt phosphorylation in wild-type arteries incubated with IRS2-deficient PVAT was comparable to incubation with wild-type PVAT after insulin stimulation (Figure 6). The failure of arteries to dilate in response to insulin after incubation with IRS2^−/−^ PVAT despite a normal Akt phosphorylation prompted us to measure eNOS phosphorylation. However, we were unsuccessful to this end due to the small amounts of eNOS protein in resistancearteries. Collectively, our results suggest that the failure of the vasodilator capacity in IRS2^−/−^ PVAT stems from changes in molecules other than adiponectin that directly affects eNOS phosphorylation. In previous publications, we showed that db/db PVAT has lost its vasodilator capacity which was accompanied by a decreased adiponectin secretion; however, adding recombinant adiponectin to db/db PVAT only partially restored insulin-induced vasodilation, suggesting that other adipokines contribute to PVAT vasodilator capacity (Meijer et al., 2013). Previs et al. have shown that IRS2-deficient adipocytes fail to attenuate lipolysis in response to insulin (Previs et al., 2000). Hence, Free Fatty Acids (FFA) are probable contributors to the impaired vasodilatory capacity of PVAT in IRS2-deficient mice. Moreover, we have previously shown (Bakker et al., 2008) that activation of PKCtheta by the FFA palmitic acid reduces insulin-mediated Akt phosphorylation (Ser(473), which is crucial in insulin-mediated activation of eNOS) while increasing ERK1/2 phosphorylation. Based on our data, we propose the following working hypothesis (depicted in Figure 7). In the healthy situation, insulin mediates vasodilation by inducing autophosphorylation of its receptor which results in phosphorylation of IRS-1 and -2 and eventually activating Akt and endothelial Nitric Oxide Synthase (eNOS) to produce NO. The PVAT secretes the necessary adipokines (represented by adiponectin) that enhance NO production in endothelial cells.IRS2-deficient PVAT fails to uncover insulin-mediated vasodilation in muscle RA. Probable mechanisms do not involve adiponectin, but rather involve direct mediators that are released from PVAT (such as FFA) that interact with eNOS to reduce NO production.

### Study limitations

In parallel with the findings of this study, there are a number of limitations that need to be considered. First, at 8 weeks of age, IRS2^−/−^ mice showed hyperinsulinemia and hyperglycemia (Table 1). Hence, it is not possible to delineate whether the dysfunction in IRS2^−/−^ PVAT is due to a direct role of IRS2 absence in PVAT signaling or due to an indirect role of the alteredmetabolic milieu. To account for this, we included young (~8–10 weeks) mice in our study which have minimal to mild whole-body metabolic dysregulation when compared to older IRS2^−/−^ mice. Previously, we have found that short term exposure of muscle RA to 10 mM of glucose does not alter their functional properties (Eringa et al., 2002). Moreover, we made our observation in an *ex vivo* set up wherein the PVAT and the arteries were incubated in a physiological buffer before the start of the experimental measurement. Hence, the indirect effects of whole-body metabolic dysregulation on PVAT phenotype in IRS2^−/−^ mice were kept to a minimum. It should be noted that while hyperglycemia-mediated effects of IRS2 deficiency cannot be fully excluded, these effects are relevant to PVAT dysfunction in DM2. Second, the adipokine secretion pattern or IRS2^−/−^ PVAT observed in this study is highlighted by a paradoxical decrease in resistin secretion. We cannot fully explain this finding, but the low inflammation in IRS2^−/−^ PVAT (Figures 4E,F) may contribute as macrophages are an important source of resistin (Qatanani et al., 2009). Third, as the PVAT in our study is missing IRS2 in all of its components (adipocytes, endothelial cells, macrophages), further experiments are needed to decipher the role of IRS2 deletion in the endothelium of PVAT. Lastly, it was not possible to measure the amount of Endothelin-1 released from the cannulated vessels in the organ bath; challenges arose in performing the *in vitro* analyses of this study due to the small amount of PVAT and small-sized arteries obtained from the mice.

In conclusion, we show in this study that in the muscle microcirculation IRS2 directly mediates insulin's vasoconstrictor effects in the vascular wall while indirectly mediating its vasodilator effects by controlling vasodilator actions of PVAT. IRS2 inactivation in PVAT abolishes its vasodilator capacity independently from adiponectin secretion or inflammation in PVAT. Future research should focus on detailed analysis of the PVAT secretome in IRS2-deficient PVAT to gain further insight into adipose tissue dysfunction in insulin-resistant individuals.

## Author contributions

AT wrote the manuscript and researched and analyzed data. WB gathered part of the data regarding the IRS2 mouse colony. HN, YS, VvH, and ES supervised, contributed to discussions, and edited the manuscript. EE researched data, supervised, contributed to discussions, and edited the manuscript. EE is the guarantor of this work and, as such, had full access to all the data in the study and takes responsibility for the integrity of the data and the accuracy of the data analysis.

### Conflict of interest statement

The authors declare that the research was conducted in the absence of any commercial or financial relationships that could be construed as a potential conflict of interest.
