# Supplementary material for: Insulin Receptor Substrate 2 Controls Insulin-Mediated Vasoreactivity and Perivascular Adipose Tissue Function in Muscle
Source: Front Physiol. 2018 Mar 23;9:245. doi: 10.3389/fphys.2018.00245 (PMC5876319; doi:10.3389/fphys.2018.00245)
Supplement: Supplementary file 1 [file DataSheet1.DOCX]

**Supplemental figure S1: Insulin-mediated vasoreactivity (A) and endothelial function (B) in resistance arteries of different mouse strains**. Sv129 genetic background modulates insulin-mediated vasoreactivity, but not endothelium-dependent vasodilation in mouse resistance arteries. Responses to different concentrations of insulin in resistance arteries of C57Bl/6 and Sv129 mice (n=6, *P<0.001) (A). Response to different concentrations of acetylcholine (ACh) in resistance arteries of C57Bl/6 and Sv129 mice (n=4).
